# Integrating Radiomics and Deep-Learning for Prognostic Evaluation in Nasopharyngeal Carcinoma

**DOI:** 10.3390/medicina61071310

**Published:** 2025-07-21

**Authors:** Irina Maria Pușcaș, Anda Gâta, Alexandra Roman, Silviu Albu, Vlad Alexandru Gâta, Alexandru Irimie

**Affiliations:** 1 Department of Otorhinolaryngology, University of Medicine and Pharmacy “Iuliu Hatieganu”, 400349 Cluj-Napoca, Romania; irinapmi@gmail.com (I.M.P.); andapstl@yahoo.com (A.G.); 2 Department of Periodontology, Faculty of Dental Medicine, Iuliu Hatieganu University of Medicine and Pharmacy, 400012 Cluj-Napoca, Romania; veve_alexandra@yahoo.com; 3 Department of Oncology, Faculty of Medicine, “Iuliu Hatieganu” University of Medicine and Pharmacy, 400347 Cluj-Napoca, Romania; vlad.gata@umfcluj.ro (V.A.G.); airimie@umfcluj.ro (A.I.)

**Keywords:** nasopharyngeal carcinoma, prognosis, radiomics, deep learning, artificial intelligence

## Abstract

Nasopharyngeal carcinoma (NPC) represents a prevalent malignant tumor within the head and neck region, and enhancing the precision of prognostic assessments is a critical objective. Recent advancements in the integration of artificial intelligence (AI) and medical imaging have spurred a surge in research focusing on NPC image analysis through AI applications, particularly employing radiomics and artificial neural network approaches. This review provides a detailed examination of the prognostic advancement in NPC, utilizing imaging studies based on radiomics and deep learning techniques. The findings from these studies offer a promising outlook for achieving exceptionally precise prognoses regarding survival and treatment responses in NPC. The limitations of existing research and the potential for further application of radiomics and deep learning in NPC imaging are explored. It is recommended that future research efforts should aim to develop a comprehensive, labeled dataset of NPC images and prioritize studies that leverage AI for NPC screening.

## 1. Introduction

Nasopharyngeal carcinoma is a common malignancy with a particularly high incidence in Southeast Asia and Southern China [1]. The cornerstone of treatment for non-metastatic NPC is radiotherapy(RT)-based modalities [2,3], which have achieved a 5-year overall survival rate exceeding 80%, largely due to significant advancements in multidisciplinary management and radiotherapy techniques [4,5]. Despite these improvements, disease progression—manifesting as locoregional recurrence or distant metastasis—remains the principal cause of treatment failure, accounting for approximately 70% of NPC-related deaths [6].

Approximately 70–80% of newly diagnosed NPC cases are categorized as locoregionally advanced (LA-NPC) [7], defined as stage III or IVA disease under the 8th edition of the American Joint Committee on Cancer (AJCC)/Union for International Cancer Control (UICC) TNM staging system [8]. For these patients, the 5-year overall survival (OS) remains suboptimal, typically around 80%, despite aggressive treatment [9].

Currently, prognosis and treatment decisions are primarily guided by the TNM staging system. However, while TNM provides essential anatomical information, it does not account for tumor biology or molecular heterogeneity. Consequently, patients within the same stage may exhibit widely divergent outcomes [10]. Standard imaging-based response evaluation criteria, such as RECIST and WHO criteria, also fall short in accurately predicting long-term outcomes like progression-free survival (PFS) [11]. Given these limitations, there is an urgent need for improved pretreatment risk stratification strategies to guide individualized treatment planning [12]. Accurate prognostic assessment at diagnosis could facilitate the classification of patients into distinct risk categories, enabling more tailored therapeutic approaches. Emerging technologies and biomarkers are being explored to enhance the prognostic utility beyond TNM staging, aiming to improve overall survival and quality of life in patients with NPC. Moreover, due to the standardized treatment protocols for NPC, which mainly include RT and chemotherapy, it could be easier to predict prognosis, compared to other head and neck pathologies which include multimodality treatment and could present variability in surgical procedures.

In recent years, AI—particularly its subfields of machine learning (ML) and deep learning (DL)—has emerged as a promising tool to enhance diagnosis, treatment planning, and prognostic prediction in oncology, including NPC [8]. AI-based models, especially those utilizing radiomics, are capable of extracting high-dimensional quantitative features from medical imaging data that are imperceptible to the human eye. These features can reflect tumor phenotype, heterogeneity, and microenvironmental characteristics [13].

Radiomics, as a subset of medical AI, enables the transformation of standard imaging modalities (CT, MRI, PET) into mineable data, facilitating non-invasive and reproducible prediction of treatment response and survival outcomes. Several studies have demonstrated the utility of radiomics in identifying high-risk NPC patients, improving early diagnostic accuracy, and optimizing personalized treatment strategies [14]. Moreover, the integration of radiomics with clinical, histopathological, and genomic data has the potential to surpass traditional staging methods in predictive accuracy [15].

While AI has yet to achieve full integration into routine clinical practice, its rapid evolution necessitates continuous reassessment of its capabilities and limitations. Radiomics-based approaches are increasingly being validated in clinical trials and offer a pathway toward more precise and individualized care for NPC patients [14].

This review highlights recent advancements and emerging opportunities offered by radiomics to enhance personalized precision medicine, with a particular focus on nasopharyngeal carcinoma (NPC). Emphasis is placed on key methodological considerations in the development and validation of radiomics-based predictive models. We further examine the integration of advanced information technologies necessary for the concurrent management of radiomic and clinical data, assess the latest imaging methodologies and deep learning algorithms, and compare recent findings in terms of predictive accuracy and clinical utility.

## 2. Materials and Methods

The authors conducted a systematic search of the MEDLINE and PubMed databases for peer-reviewed literature, employing relevant MeSH terms including “radiomics”, “FDG-PET”, “MRI”, “CT”, “imaging”, in conjunction with “nasopharyngeal carcinoma” or “NPC”, and “deep learning prediction” or “artificial intelligence”. Only articles published in the English language were considered. The search encompassed studies with varying levels of evidence, including randomized controlled trials, cohort studies, and case-control studies. Inclusion criteria were restricted to studies employing artificial intelligence techniques and radiomics for the purpose of predicting disease progression or clinical outcomes in patients with nasopharyngeal carcinoma.

## 3. Results and Discussions

### 3.1. The Principle of Radiomics

Radiomics was first proposed by Lambin in 2021 [16] and it encompasses a set of computational methodologies or algorithms aimed at extracting a high-dimensional set of quantitative features from radiological medical images [13]. Radiomics transforms imaging data into a high-dimensional, mineable feature space by employing numerous automated algorithms to extract quantitative descriptors. This process enables the identification of tumor characteristics that may be imperceptible to the human eye, thereby facilitating a comprehensive, objective characterization of the tumor phenotype [10,13,17].

Radiomic statistical features are typically categorized into first-order, second-order, and higher-order features [18,19]. First-order features quantify the distribution of voxel intensities within the region of interest, without accounting for spatial relationships between voxels. Second-order features, commonly referred to as texture features, characterize the spatial relationships and statistical dependencies between neighboring voxels, thereby providing insights into intratumoral heterogeneity. High-order features are derived by applying filter grids to the image, enabling the extraction of repeated or non-repeated patterns [16].

A radiomics analysis typically comprises four key phases: (1) data acquisition and curation, (2) feature extraction and selection, (3) model development and (4) validation. Below we report a typical step-by-step radiomics workflow.

#### 3.1.1. Data Acquisition and Curation

Radiomic analysis typically begins with the selection of an appropriate imaging modality (e.g., CT, MRI, PET), the delineation of the region or volume of interest (ROI or VOI), and the definition of a specific predictive endpoint that addresses a clinically relevant question. In oncological research, radiomic evaluation is most commonly applied to the entire primary tumor, with extracted features subsequently correlated with clinical outcomes such as survival rates or tumor response to treatment [19]. Additionally, radiomic analysis can be extended to tumor subregions (habitats), metastatic lesions, and even to normal tissues to explore spatial and biological heterogeneity. Standardization of imaging protocols is essential to reduce variability and ensure the reliability of radiomic analyses [13,20]. Despite increasing recognition of its importance, the absence of uniform imaging acquisition protocols continues to represent a major challenge in the field. This lack of standardization contributes to reduced reproducibility and limits the comparability of findings across studies. To address these concerns, there is a pressing need for clear, widely adopted guidelines on imaging protocol implementation and reporting. In the meantime, careful evaluation and, when possible, harmonization of imaging datasets according to established radiomics principles remain critical steps in improving study quality [21].

Segmentation represents the initial and essential step in radiomics analysis, and it can be performed manually by experienced radiologists or clinicians, or through (semi-)automated techniques [14,22]. Each approach presents specific advantages and limitations, with the optimal method depending on the clinical context and imaging characteristics. In general, automated segmentation offers greater reproducibility and efficiency compared to manual delineation [23]. This step is critical, as it defines the set of voxels within the image that will be subjected to further radiomic analysis.

#### 3.1.2. Feature Extraction and Selection

At the core of radiomics lies the extraction of quantitative features from medical images to characterize volumes of interest (VOIs) [14]. Hand-crafted radiomic features are commonly categorized into five groups: (1) size- and shape-based descriptors, (2) intensity histogram-based features, (3) features describing spatial relationships between voxels, (4) features derived from filtered images, and (5) fractal-based metrics [24]. The values of these features are highly influenced by various image preprocessing steps, including filtering, intensity discretization, and image reconstruction parameters [14]. To ensure the harmonization of radiomic features and model outputs, all sources of variability must be carefully considered, and detailed specifications should accompany each model to promote transparency and reproducibility [21].

The true potential of radiomics lies in its integration with non-image-based clinical data to form a unified dataset linked to a defined prediction endpoint. This integrative approach facilitates the exploration of potential correlations among diverse features. However, radiomic features that exhibit strong correlation with routinely available clinical variables—such as tumor stage or patient age—may offer limited incremental value in predictive modeling.

#### 3.1.3. Model Development

The selection of an appropriate machine learning algorithm represents a critical step in the modeling process, since the choice of technique has been shown to significantly influence predictive performance in radiomics [14]. Ideally, multiple algorithms should be applied and systematically compared to identify the most effective approach, with each implementation thoroughly documented [25]. Therefore, in an optimal scenario, multiple modeling approaches should be employed and systematically compared, with each method’s implementation comprehensively documented to ensure transparency and reproducibility. Another essential consideration in selecting a modeling methodology is its replicability by other researchers, in alignment with the principles of responsible and transparent research and innovation [14].

#### 3.1.4. Validation

Validation techniques are essential for evaluating the generalizability of predictive models in radiomics. Optimal validation involves both internal and external procedures, with performance metrics transparently reported and compared to ensure robustness and reproducibility [14]. They address the critical question of whether a model reliably predicts outcomes across the broader target patient population or only within the specific subset used for model development. Model performance is typically assessed in terms of discrimination and calibration. Discrimination reflects the model’s ability to distinguish between outcome classes and is commonly quantified using concordance statistics. For binary outcomes, metrics such as the receiver operating characteristic (ROC) curve and the area under the ROC curve are employed [14]. The AUC is directly related to model sensitivity and specificity, representing the probability that a randomly selected patient with the outcome is assigned a higher predicted probability than a randomly selected patient without the outcome. Calibration, on the other hand, measures the agreement between predicted probabilities and observed outcomes. It can be evaluated using calibration plots, calibration-in-the-large, calibration slope, and the Brier score, which represents the mean squared prediction error and provides an overall measure of predictive performance [26]. To ensure reproducibility and transparency, the statistical methods applied to both training and validation datasets should be reported in detail.

### 3.2. Artificial Intelligence Technologies

Recent advancements in AI have enabled the automated analysis and interpretation of complex datasets. AI is increasingly utilized in data-intensive medical disciplines such as oncology, radiology, and pathology, where precise image analysis is essentia [27]. Traditionally, the evaluation of head and neck pathologies has relied on visual assessment of medical images by clinicians—a process that is inherently subjective and dependent on individual expertise. In contrast, AI offers a quantitative approach, capable of automatically identifying and interpreting imaging features [28]. By incorporating both traditional ML and DL techniques, AI enhances diagnostic accuracy, accelerates image interpretation, and significantly reduces clinician workload [29]. Traditional ML algorithms represent a foundational AI approach in medical imaging, relying heavily on manually engineered features that are mathematically defined and computationally quantifiable (e.g., tumor texture characteristics). These features are input into ML models to assist clinicians in patient classification and clinical decision-making. Commonly used ML algorithms include k-nearest neighbors (KNN), support vector machines (SVM), and random forests (RF), among others. In radiology, these methods are frequently employed to transform image data into numerical feature vectors through dedicated image processing techniques [28]. Predictive models are subsequently constructed using these vectors to extract clinically relevant information from the imaging data. Radiomics, which applies this methodology, has been investigated in several small-scale retrospective studies aimed at predicting tissue subtypes, treatment response, patient prognosis, and other tumor-related characteristics from medical images.

Deep learning, a subset of machine learning, is based on neural network architectures inspired by the human brain. Unlike traditional ML models, which require manual definition and extraction of image features—making their performance heavily dependent on feature quality—DL algorithms can autonomously learn and extract features directly from raw data [30]. This data-driven approach enables DL models to perform complex tasks such as image classification and processing with enhanced adaptability and scalability. Common DL architectures used in medical image analysis include artificial neural networks (ANN), deep neural networks (DNN), convolutional neural networks (CNN), and recurrent neural networks (RNN) [31]. Among these, CNNs are currently the most widely adopted architecture in the field. A CNN typically comprises multiple layers, including convolutional, pooling, and fully connected layers, which process image pixels through feature extraction and hierarchical abstraction. Deep convolutional neural networks (DCNNs), which include more convolutional layers and parameters, are optimized for large-scale datasets. The U-Net architecture, which employs fully convolutional layers and image augmentation strategies, achieves high accuracy even with limited training data. RNNs are particularly suited for analyzing sequential or time-series data [32]. Each DL architecture exhibits distinct advantages and is suited to specific medical imaging applications.

### 3.3. Advances of Prediction in NPC Based on Radiomics and Deep Learning

#### 3.3.1. MRI-Radiomics and Deep-Learning Prediction in NPC

One of the largest cohorts investigating prognostic prediction in NPC using integrated MRI radiomics and deep learning methodologies was reported by Zhong et al. [33]. The study included 1872 patients diagnosed with stage T3N1M0 NPC across four centers in China. The research team based in Beijing developed and validated a multi-task nomogram capable of noninvasively predicting prognosis and recommending optimal treatment strategies for patients undergoing various therapeutic regimens. Their findings highlighted the critical importance of incorporating treatment variables into prognostic models, as evidenced by a marked improvement in predictive performance when treatment factors were included. Specifically, the single-task radiomic model achieved a C-index of 0.759, whereas the multi-task model—combining both prognostic and predictive scores—demonstrated a superior C-index of 0.836. Notably, the investigators observed that high-risk patients with poor survival outcomes typically presented with more aggressive primary tumors and a diminished peritumoral immune response. Conversely, low-risk patients with favorable prognosis exhibited less aggressive tumors and a heightened peritumoral immune response.

A comparable nomogram was developed by Xue Ming et al. [34], integrated radiomic features with clinical parameters. This model demonstrated robust performance in the validation cohort, achieving a C-index of 0.751 [95% CI: 0.639–0.863] for disease-free survival (DFS) and 0.845 [95% CI: 0.752–0.939] for OS. The incorporation of radiomic features significantly enhanced predictive accuracy, yielding improvements in the C-index of 0.029 for DFS and 0.107 for OS compared to models based solely on clinical features.

While most radiomics-based prognostic models demonstrate improved performance when combined with clinical data, the study by Hao-Jiang Li et al. [35]. reported an exception. In their analysis of distant metastasis-free survival (DMFS) prediction, radiomics-only models outperformed those that integrated both radiomic and clinical features. Specifically, the radiomics models achieved concordance indices ranging from 0.645 to 0.722, compared to lower C-indices of 0.605 to 0.678 for the combined clinical-radiomics models.

A research group from Guangzhou (Da-Feng et al. [36]) developed an MRI-based radiomic signature that demonstrated superior prognostic performance compared to conventional clinical indicators in predicting overall survival in patients with locally recurrent nasopharyngeal carcinoma. The model achieved concordance indices of 0.729, 0.718, and 0.731 in the training, internal validation, and external validation cohorts, respectively. Furthermore, Da-Feng et al. [36]. correlated this radiomic signature with tumor immune heterogeneity, highlighting its potential utility in refining prognostic stratification and informing personalized treatment strategies.

With regard to the MRI-based imaging characteristics of NPC, several studies have highlighted the relevance of incorporating morphological features into predictive algorithms. Li et al. [37] emphasized the prognostic significance of the peritumoral region on MRI. In this context, a research group from Wuhan developed prediction models based on conventional tumor segmentation and further extended the segmentation to include the peritumoral region at varying distances. The average AUC values for deep learning models trained on the original tumor segmentation and on segmentations expanded by 5, 10, 20, 40, and 60 pixels were 0.717 ± 0.043 for the original segmentation and 0.760 ± 0.010, 0.768 ± 0.018, 0.802 ± 0.013, 0.782 ± 0.039, and 0.753 ± 0.014 for each expansion, respectively. The optimal model performance was achieved with a 20-pixel peritumoral expansion. These findings support the notion that the peritumoral region contains prognostically valuable information, and its inclusion can significantly enhance the accuracy of MRI-based prognostic models in NPC.

Since prediction based on radiomics is still in its infancy, there is no clear protocol regarding the best-performing MRI incidence or technique used. Hailin Li et al. [38] developed an integrated prognostic model combining conventional magnetic resonance (MR) imaging with dynamic contrast-enhanced MR (DCE-MR) radiomic features. The inclusion of DCE-MR data significantly improved predictive performance compared to models based solely on MR or DCE-MR imaging. In the test cohort, the combined model achieved a C-index of 0.808, outperforming the MR-only and DCE-MR-only models, which achieved C-indices of 0.729 and 0.731, respectively. These findings demonstrate the potential of integrating MR and DCE-MR radiomic features to enhance the accuracy of prognostic assessments in patients with nasopharyngeal carcinoma.

In a similar investigation Qiyi Hu et al. [39], another research group assessed the prognostic utility of radiomic analysis derived from readout-segmented echo-planar diffusion-weighted imaging (RESOLVE-DWI), a technique that quantitatively characterizes the diffusion motion of water molecules. Their findings demonstrated that the random forest (RF) model based on radiomic features extracted from DWI and apparent diffusion coefficient (ADC) sequences achieved an average cross-validated area under the curve of 0.80. In contrast, RF models constructed using conventional MRI sequences—including DWI, ADC maps, T2-weighted imaging (T2WI), and contrast-enhanced T1-weighted imaging—yielded a lower AUC of 0.72. These results suggest that RESOLVE-DWI-based radiomics may provide superior prognostic performance in nasopharyngeal carcinoma.

#### 3.3.2. CT Scan Radiomics Prediction in NPC

Radiomics and deep learning models for prognostication in nasopharyngeal carcinoma (NPC) have rarely utilized computed tomography (CT) as the primary imaging modality for model construction (as noted by Intarak et al. [40]). However, a research group from Thailand investigated a multimodal approach by combining magnetic resonance radiomics, contrast-enhanced CT imaging, and clinical variables. Their findings indicated that this integrative model significantly improved predictive performance for OS, PFS, and DMFS compared to models based solely on conventional clinical parameters. Specifically, the combined radiomics-clinical model achieved concordance indices ranging from 0.788 ± 0.066 to 0.848 ± 0.079, in contrast to 0.745 ± 0.082 to 0.766 ± 0.083 for clinical models alone, underscoring the added prognostic value of multimodality radiomic data.

#### 3.3.3. PET/CT-Radiomics and Deep Learning Prediction in NPC

Certain authors, such as Bingxin Gu et al. [41] have noted that conventional imaging modalities, including CT and MRI, are limited to providing anatomical information about the tumor. In contrast, multimodal imaging techniques such as positron emission tomography/computed tomography (PET/CT) offer a more comprehensive assessment by integrating anatomical detail from CT with metabolic activity data from PET, thereby enabling a more accurate evaluation of tumor characteristics. Certain quantitative parameters derived from PET/CT imaging—such as maximum standardized uptake value (SUVmax), mean standardized uptake value (SUVmean), metabolic tumor volume (MTV), and total lesion glycolysis (TLG)—have demonstrated prognostic relevance in various studies [42]. However, these quantitative biomarkers fail to capture the spatial heterogeneity of tumors, thereby limiting the precision and overall accuracy of prognostic prediction.

Recent advances in radiomics have enabled the extraction of high-throughput, quantifiable image-based features, thereby facilitating the assessment of intratumoral heterogeneity in PET imaging [13].

Lv WB et al. [43] extracted radiomic features from both the PET and CT components of baseline PET/CT imaging, enabling quantitative characterization of intratumoral heterogeneity. These features were shown to provide complementary prognostic information in patients with NPC. The integration of PET and/or CT-derived radiomic features with clinical parameters resulted in improved predictive performance for patient outcomes compared to models based solely on radiomics or clinical data. This enhancement was particularly evident in the subset of patients with locoregionally advanced disease, across both training and validation cohorts. Interestingly, in a related study on PET/CT radiomics, Xu et al. [44] demonstrated that subregional imaging biomarkers exhibited superior prognostic performance compared to whole-tumor biomarkers in predicting PFS, with concordance indices of 0.69 and 0.58, respectively.

Moving a step forward, Binxin Gu et al. [45] developed a three-dimensional (3D) PET/CT end-to-end deep learning model for the prediction of 5-year progression-free survival, demonstrating superior performance compared to conventional radiomics approaches. Their multimodal deep learning radiomics model, which integrates both PET and CT imaging data, achieved higher prognostic accuracy, AUC values of 0.842 ± 0.034 in the internal cohort and 0.823 ± 0.012 in the external cohort. These results outperformed the best-performing conventional radiomics model, which yielded AUCs of 0.796 ± 0.033 and 0.782 ± 0.012, respectively. Additionally, the multimodal DLR model surpassed the performance of single-modality DLR models, which were based solely on PET (AUC = 0.818 ± 0.029 and 0.796 ± 0.009) or CT (AUC = 0.657 ± 0.055 and 0.645 ± 0.021).

These findings may be contextualized by comparison with a study conducted by Zhang et al. [46], which also targeted similar clinical endpoints (DMFS and PFS) using comparable evaluation metrics (AUC). Despite incorporating additional clinical variables such as EBV DNA levels and treatment regimens, Zhang et al.’s two-dimensional (2D) MRI model demonstrated inferior performance (AUC = 0.795) compared to Bingxin Gu Bingxin Gu et al.’s 3D model (AUC = 0.842) [41].

Gu et al. subsequently expanded their research in a multicenter study published the following year. In this study Bingxin Gu et al. [45], their multi-task deep learning radiomics (MTDLR) nomogram achieved concordance indices of 0.818 (95% CI: 0.785–0.851) in the training cohort, 0.752 (95% CI: 0.638–0.865) in the internal validation cohort, and 0.717 (95% CI: 0.641–0.793) in the external validation cohort for predicting progression-free survival. Corresponding AUC values were 0.859 (95% CI: 0.822–0.895), 0.769 (95% CI: 0.642–0.896), and 0.730 (95% CI: 0.634–0.826), respectively. These results demonstrated a statistically significant improvement over conventional radiomics-based nomograms in prognostic prediction and managed to stratify low and high-risk patients.

#### 3.3.4. Multi-Modality Radiomics and Deep Learning Prediction in NPC

Kulanthaivelu R et al. [47] developed prognostic models for patients with NPC by integrating radiomic features derived from PET/CT and MRI, along with baseline clinical parameters. Their findings demonstrated that the combination of PET/CT- and MR-based radiomics significantly enhanced the prediction of overall survival and progression-free survival. Notably, the combined model yielded high predictive performance, with AUC values of 0.96 for OS and 0.86 for PFS, indicating the added value of multimodal imaging in prognostic stratification.

Another complex prediction model was developed by Zhichao Zuo and colleagues [48] utilizing three machine learning algorithm combinations, incorporating 33 features derived from clinicopathological data, hematologic biomarkers, and MRI findings. Their multi-layered decision prognostic system (MLDPS) demonstrated strong calibration and discriminative performance in predicting PFS among patients with non-metastatic nasopharyngeal carcinoma. The model’s predictive accuracy improved progressively with the inclusion of additional data types, with the concordance index increasing from 0.58 for the clinical model to 0.639 for the MRI-based model, and reaching 0.662 for the combined model. These findings highlight the potential value of integrating non-invasive hematologic markers into radiomics-based prognostic frameworks to enhance outcome prediction in NPC. Details regarding the imaging technique, cohort size, feature selection and model employed are summarized in Table 1.

## 4. Conclusions

We are currently witnessing a transformative shift in medical practice through the integration of artificial intelligence. In the context of nasopharyngeal carcinoma, prognostic capabilities have notably improved with the application of deep learning and radiomics techniques. Despite these advancements, the field remains complex and heterogeneous, and standardized protocols defining the optimal imaging modalities and the most effective deep learning algorithms have yet to be established. Nevertheless, existing evidence suggests that the inclusion of a broader spectrum of input data enhances the predictive accuracy of these models.

## Figures and Tables

**Table 1:** Studies predicting prognosis of NPC patients, using radiomics and deep learning.

Author	Imaging Technique	Cohort/Sample Size	Feature Selection	Modeling	Endpoint	Algorithm Performance/Model Evaluation
Peng, H. et al., 2019 [49]	PET/CT	707 patients	LASSO Cox regression Rad score	Nomogram 4 DCNN	DFS, OS, DMFS, LRRFS	C-index: 0.754 training, 0.722 validation
Ming, X. et al., 2019 [34]	MRI	303 patients	Non-negative matrix factorization	Chi-squared test, nomogram	DFS, OS, DMFS, LRFS	DFS C-index: 0.751 OS C-index: 0.845
Mao, J et al., 2019 [50]	MRI	79 patients	Cox regression	Kaplan-Meier	PFS	AUC: 0.825 C-index: 0.794
Yang, K et al., 2019 [51]	MRI, clincial features	224 patients	LASSO regression	Univariate Cox regression Nomogram	PFS	C-index: 0.811
Lv, W. et al., 2019 [43]	PET/CT	128 patients	MATLAB R2016a package	Univariate analysis with FDR correction Spearmans’s correlation Cox regression	PFS	C-index: 0.71 training, 0.76 validation
Xu, H. et al., 2020 [44]	PET/CT	128 patients	Cox regression Pearson correlation	Kaplan-Meier Cox regression	PFS	C-index: 0.69 for biomarker S3, 0.58 for whole tumor
Shen, H et al., 2020 [52]	MRI	327 patients	LASSO regression	Kaplan-Meier	PFS	C-index: 0.749 training, 0.836 validation
Bao, D. et al., 2021 [53]	MRI	199 patients	Wilcoxon signed-rank test LASSO Cox regression	Rad Score Nomogram Cox regression	PFS	AUC: 0.604 training, 0.732 validation
Kim, M-J. et al., 2021 [54]	MRI, clinical features	81 patients	LASSO Cox regression	Univariate Cox regression Nomogram	PFS	iAUC: 0.76 training, 0.81 validation
Yan, C et al., 2021 [55]	CT, clinical features	311 patients	LASSO regression	Nomogram DeLong test	PFS	C-index: 0.873
Kang, L, et al. 2021 [56]	MRI	243 patients	LASSO Pearson correlation SMOTE algorithm	Univariate and multivariate analysis	PFS	AUC: 0.773 training, 0.852 validation
Zhu C et al., 2021 [57]	CT scan	156 patients	LASSO regression *t*-test	Rad score Nomogram	Predict recurrence	C-index: 0.931 training, 0.799 validation
Hu, Q. et al., 2022 [39]	MRI-DWI	154 patients	Manual segmentation Relative feature extraction	Logistic regression, Naive Bayes Random Forest, XGB Classifier	Recurrence and metastasis	AUC: 0.80 (95% CI: 0.79–0.81)
Gu, B. et al., 2022 [41]	PET/CT 3d CNN	257 patients	DCNN	End-to-end multi-modality DLR model	5-year PFS	AUC: 0.842 ± 0.034 training, 0.823 ± 0.012 validation
Kulanthaivel R. et al., 2022 [47]	PET/CT, MR, clinical features	124 patients	Semi-automatic	Cox regression Spearman’s correlation	OS, PFS	AUC: 0.86 for 21 months PFS and 0.96 for 24 months OS
Zeng, F. et al., 2022 [58]	MRI, clinical features	110 patients	Pearson correlation, univariate Cox, LASSO	Logistic regression Nomogram	PFS	C-index: 0.814
Li, H-J. et al., 2022 [35]	MRI	260 patients	Pearson correlation Cox regression	Kaplan-Meier Cox regression	DMFS	C-index: 0.722 (95% CI = 0.632–0.811)
Intarak et al., 2022 [40]	CT	197 patients	Cox regression LASSO	Cox regression	PFS, OS, DMFS	AUC: 0.747 ± 0.062 C-index: 0.767 ± 0.074
Gu, B., et al., 2023 [41]	FDG-PET/CT, clinical features	886 patients	LASSO Cox regression	MTDLRN integrating DeepMTS-score AutoRadio-score	PFS	C-index: 0.818 training, 0.752 validation AUC: 0.859 training, 0.769 validation
Sun, M-X. et al., 2023 [59]	MRI, clinical features	120 patients	LASSO regression	Random Forest, Harell’s concordance index, Nomogram, Kaplan-Meier	PFS	C-index: 0.953
Long, Z. et al., 2023 [60]	FDG PET-CT	173 patients	LASSO regression Cox model	Harell’s concordance index, Kalpan-Meier, Log-rank	OS	C-index: 0.779 training, 0.744 validation
Li, H. et al., 2023 [38]	DCE-MR	434 patients	LASSO Cox regression	Pearson correlation Kaplan-Meier	PFS	C-index: 0.812 training, 0.808 validation
Li, S. et al., 2023 [37]	MRI- peritumoral region	381 patients	Deeplab v3 U-Net	Neural network	Prognosis	Dice coefficients: 0.741 and 0.737
Khongwirotphan, S. et al., 2024 [61]	CT/MRI	183 patients	Cox regression	Cox regression	OS, PFS, DMFS	OS: C-index: 0.879 training, 0.827 test PFS: C-index: 0.721 training, 0.722 test DMFS: C-index: 0.802 training, 0.779 test
Dang, L. et al., 2024 [62]	MRI	276 patients	Cox regression	Kaplan-Meier Nomogram	PFS	AUC: 0.66 training, 0.717 validation
Xi, YZ. et al., 2024 [63]	MRI	313 patients	LASSO regression Rad score	Logistic regression Nomogram	5 year PFS	AUC: 0.83 training, 0.81 validation
Lin, D-F., et al., 2024 [36]	MRI	921 patients	Cox regression Nomogram	Kaplan-Meier Cox regression	OS	C-index: 0.729 training, 0.718 internal validation, 0.731 external validation

Abbreviation: Deep Convolutional Neural Network (DCNN), Disease-free survival (DFS), Overall survival (OS), Distant metastasis-free survival (DMFS), Benjamini–Hochberg false discovery rate (FDR), Loco-regional relaps-free survival (LRRFS), Progression-free survival (PFS), Integrated time-dependent area under the curve (iAUC), Convolutional neural network (CNN), Multi task deep learning-based radiomic nomogram (MTDLRN), Dynamic contrast enhanced (DCE).

## Data Availability

Data available on request.

## Abbreviations

The following abbreviations are used in this manuscript:

NPC	Nasopharyngeal carcinoma
AI	Artificial intelligence
DL	Deep learning
ML	Machine learning
CT	Computed tomography
MRI	Magnetic resonance imaging
PET	Positron emission tomography
RT	Radiotherapy
PFS	Progression-free survival
DMFS	Distant metastasis-free survival
OS	Overall survival
DFS	Disease-free survival
